# One Century of Change: Stronger Diversity Decline in Lowland Than in Mountain Grasslands in Central Europe

**DOI:** 10.1111/gcb.70529

**Published:** 2025-10-03

**Authors:** Stefan Widmer, Susanne Riedel, Manuel Babbi, Felix Herzog, Thomas Wohlgemuth, Michael Kessler, Jürgen Dengler

**Affiliations:** ^1^ Vegetation Ecology Research Group, Institute of Natural Resource Sciences ZHAW Zurich University of Applied Sciences Wädenswil Switzerland; ^2^ Department of Systematic and Evolutionary Botany University of Zurich Zurich Switzerland; ^3^ Agricultural Landscapes and Biodiversity Agroscope Zurich Switzerland; ^4^ Swiss Federal Institute for Forest Snow and Landscape Research WSL Birmensdorf Switzerland

**Keywords:** biodiversity change, Central Europe, climate change, elevational gradient, eutrophication, grassland, land use change, long‐term vegetation dynamics, Switzerland, vegetation resurvey

## Abstract

Quantitative long‐term assessments of the extent and direction of biodiversity change due to anthropogenic environmental change are challenging because representative baseline data older than a few decades are very rare. This is also the case for grasslands in temperate Europe, which can harbour high species diversity at small spatial scales, but have undergone strong and varied changes, particularly in relation to the agricultural intensification that peaked in the middle of the last century. We resampled 416 historical vegetation records (originally sampled between 1884 and 1931) of 0.09 m^2^ from grasslands across Switzerland at a wide range of elevations (300–2500 m) and moisture levels to assess the changes in taxonomic, functional, and phylogenetic diversity, as well as community characteristics, and tested whether the magnitude of change depended on elevation. We found severe declines in alpha, beta, and gamma taxonomic diversity over the last century, with species richness being on average 26% lower in the resurvey plots than in the historical plots. Functional and phylogenetic alpha diversity were also lower in the resurvey plots than in the historical plots, although the differences were less pronounced. The loss in all three diversity metrics decreased with elevation. This was linked to stronger increases in nutrient‐demanding, mowing‐tolerant, and competitive species, particularly grasses (Poaceae), at lower than at higher elevations. This elevational pattern reflects the strong influences of land use and eutrophication, which are more pronounced at lower elevations. By contrast, the effect of global warming on vegetation has so far been subordinate. The smaller diversity changes at higher elevations offer the potential to maintain a high proportion of the historical plant diversity in mountain grasslands.

## Graphical abstract

Written Summary: We resampled 416 historical vegetation records (originally sampled between 1884 and 1931) from grasslands across Switzerland at a wide range of elevations and moisture levels. We found severe declines in alpha, beta, and gamma taxonomic diversity as well as functional and phylogenetic alpha diversity over the last century. The biodiversity loss was strongest in the lowlands and decreased with elevation. This was linked to stronger increases in nutrient‐demanding, mowing‐tolerant, and competitive species, particularly grasses, at lower than at higher elevations, reflecting the strong influences of land use and eutrophication, which are more pronounced at lower elevations.

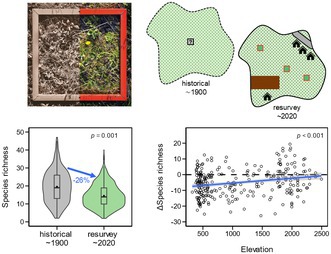

## 1 Introduction

The ongoing global decline in biodiversity is well documented and widely recognized (Ceballos et al. 2015; Dirzo and Raven 2003; IPBES 2019). In Europe and other industrialized regions of the world, land use intensification, which rapidly increased in the second half of the 20th century (Green 1990; Lachat et al. 2010; Matson et al. 1997), is considered to be one of the main causes of this loss (IPBES 2019; Jaureguiberry et al. 2022; Kleijn et al. 2009). However, despite general consensus on biodiversity decline, robust quantitative assessments spanning the full extent of the past century hardly exist. This is largely due to the paucity of baseline data extending back more than a few decades, especially across large spatial scales (Jandt, Bruelheide, Berg, et al. 2022; Kapfer et al. 2017). Consequently, most long‐term assessments rely on indirect and coarse indicators such as changes in land cover or habitat extent (e.g., Butchart et al. 2010; Lachat et al. 2010; Sala 2000), whose link to species diversity is complex and non‐linear.

Among the habitat types most strongly affected by land‐use change in Europe are semi‐natural grasslands, now classified among the most threatened ecosystems of the continent (Janssen et al. 2016). These grasslands are the product of millennia of low‐intensity agricultural practices dating back to the Neolithic (Hejcman et al. 2013). In contrast to intensively managed grasslands, they have experienced minimal fertilizer input and no systematic reseeding (Dengler et al. 2020). As a result, they support exceptionally high species richness at fine spatial scales—plot‐level vascular plant diversity in these systems ranks among the highest globally (Wilson et al. 2012). In addition to hosting numerous endemic and specialist species, semi‐natural grasslands play a significant role in maintaining ecosystem functions and services (Dengler et al. 2014).

At coarse spatial scales, such as countries, regions, or geographic grid cells, the decline of grassland plant diversity is well documented quantitatively (Eichenberg et al. 2021) and qualitatively through Red Lists (Bornand et al. 2016). However, at fine spatial scales (*α* diversity) where many of the potential drivers act on plant diversity, the picture is inconclusive to date. While previous resurvey studies in Central European grasslands generally found clear shifts in species composition, many of them reported no change or even an increase in species richness (Häberlin and Dengler 2025; Klinkovská et al. 2025; Schwaiger et al. 2022). Apart from the effects of different spatial scales (regional vs. local), a major reason for these contrasting findings is probably that the original vegetation records that were resurveyed and that could provide reliable estimates of plant community change (Kapfer et al. 2017) typically date back only one to seven decades (e.g., Diekmann et al. 2019; Jandt, Bruelheide, Berg, et al. 2022). Thus, most resurvey studies rely on baseline data from during or after the Third Agricultural Revolution (Green 1990; Lachat et al. 2010; Matson et al. 1997; Weber et al. 2004; Wood et al. 2017). Longer‐term resurvey studies of vegetation plots are extremely rare and hitherto restricted to spatially limited sites like mountain tops (Steinbauer et al. 2018) or a local grassland experiment (Silvertown et al. 2006), and thus hardly generalizable to widespread ecosystems (Verheyen et al. 2017). Lacking representative baseline data prior to the intensive agricultural transformation limits our understanding of longer‐term changes in species diversity, community composition, and ecological strategies and could lead to an over‐optimistic picture of the current state of grassland biodiversity in Europe.

The vast majority of studies on diversity change of vegetation focus on taxonomic alpha diversity, mostly species richness, thus failing to provide a comprehensive picture of the nature of change and its implications. The few studies that also considered the beta component of diversity, i.e., the spatial turnover in species composition, often found that a local or regional increase in plant diversity was associated with a spatial homogenization, i.e., a decrease in beta diversity (Bühler and Roth 2011; Finderup Nielsen et al. 2019). Even more rarely, studies on long‐term dynamics in plant diversity consider phylogenetic and functional diversity in addition to taxonomic diversity (but see Gillet et al. 2016). Thus, our understanding of how humans altered these two aspects of biodiversity remains limited.

Another major limitation of previous work on anthropogenic plant diversity change is the underrepresentation of elevational gradients in the studies. While contemporary species richness often peaks at mid‐elevations (Descombes et al. 2017; Güsewell et al. 2012; Wohlgemuth et al. 2008), this relationship is known to vary with spatial scale (Nogués‐Bravo et al. 2008; Rahbek 2005). Whether the observed elevational richness patterns are primarily shaped by natural abiotic gradients or have been substantially modified by anthropogenic pressures over time remains unresolved (Nogués‐Bravo et al. 2008).

In this study, we leverage a unique historical dataset of systematically surveyed vegetation plots in grasslands across Switzerland, originally recorded between 1884 and 1931 using consistent methods (Riedel et al. 2023). This dataset offers an unprecedented opportunity to quantify long‐term changes in plant diversity and composition comprehensively across a broad spatial and elevational extent. Considering that our baseline data are from before the Third Agricultural Revolution, which was much more impactful at lower elevations, we tested the following main hypotheses: (i) over this long period all components (*α*, *β*, *γ*) and aspects of diversity (taxonomic, phylogenetic, and functional) have declined; (ii) the plant community has shifted toward species indicative of higher nutrient availability and management intensity; and (iii) the magnitude of these changes varies with elevation, with lowland areas exhibiting more pronounced transformations than higher‐elevation sites.

## 2 Methods

### 2.1 Historical Vegetation Records and Relocation

For the resurvey, we used the “Historic Square Foot Dataset” (Riedel et al. 2023) consisting of 580 vegetation plots of 0.09 m^2^ that were sampled across Switzerland between 1884 and 1931. The researchers who recorded the historical plots aimed at characterizing the grassland types in Switzerland. Therefore, the historical plots cover a wide range of grassland types along the elevation and soil moisture gradients (Riedel et al. 2023). The historical plots were sampled by cutting a 0.3 × 0.3 m (“square foot”) section of the sod, followed by measuring the aboveground dry mass of each species in the lab.

**FIGURE 1:**
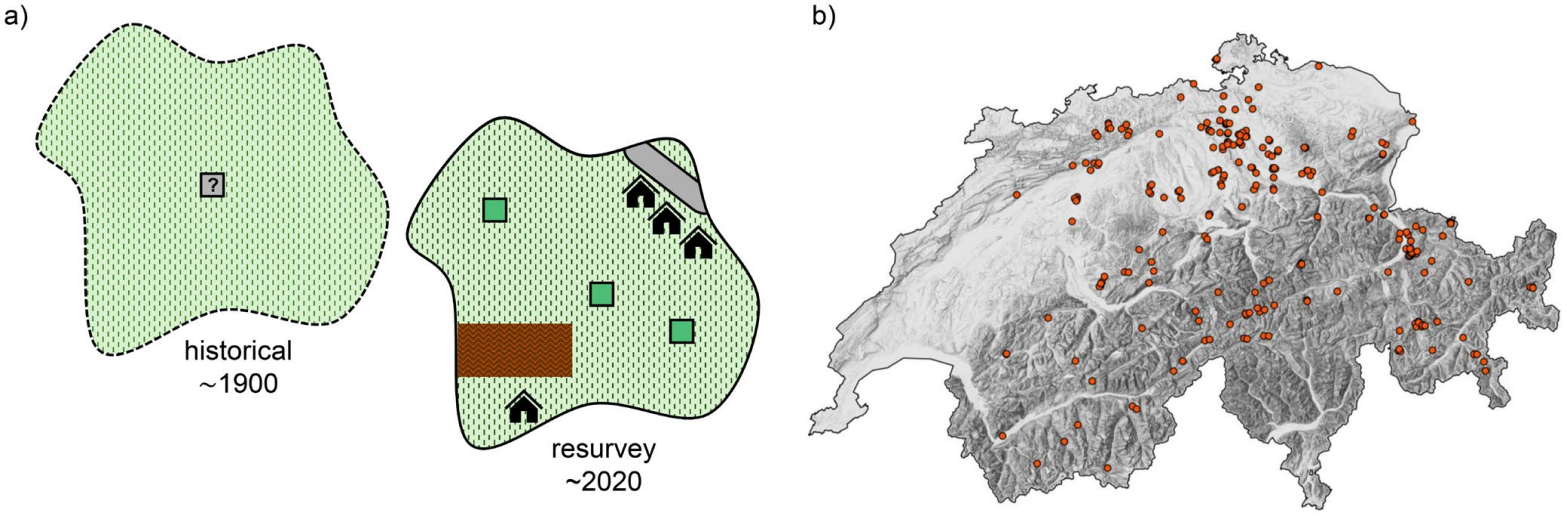
(a) For each historical vegetation plot we defined a potential area in which the historical plot most likely was located (left). Based on the size of the area still covered with grassland, we sampled 3–5 randomly distributed resurvey plots (right). (b) Location of the “potential areas” in Switzerland (background map © swisstopo). Map lines delineate study areas and do not necessarily depict accepted national boundaries.

For the resurvey, for each historical plot, we defined a potential area where the historical plot was most likely located based on the description of the location in the old records. These records normally included the name of the village or town and the elevation, and often additional information such as toponyms, aspect, or distance and direction to a certain landmark or farm (Figure 1). Historical vegetation‐plot records with identical or very similar location information were assigned to the same potential area, resulting in 377 potential areas for the original 580 plots. Further details about the “Historic Square Foot Dataset”, including the relocation of the historical plots, can be found in Riedel et al. (2023).

### 2.2 Vegetation Resurvey

#### 2.2.1 Sampling Design

Due to changes in land use, some parts of the potential areas were no longer covered by grassland during our resurvey in 2021 and 2022. We excluded these non‐grassland areas, such as built‐up areas and forests, from the potential areas based on the land cover layer of the Swiss topographic landscape model (https://www.swisstopo.admin.ch/en/landscape‐model‐swisstlm3d) using ArcGIS Pro 2.6.3 (ESRI). In addition, we excluded arable land by using geodata containing the land use of farmland (https://www.geodienste.ch/services/lwb_nutzungsflaechen, 2021). In 19 potential areas, no grassland was left, leaving 358 potential areas representing 542 historical vegetation plots.

In each potential area, we sampled 3–5 randomly distributed resurvey plots to account for the error due to imprecise relocation (Figure 1). The relocation error will increase the Type II errors, i.e., make our statistical tests conservative, but should not lead to any bias in the data if the new plots are randomly placed as in our case (Boch et al. 2019; Kapfer et al. 2017). The relocation error depends on spatial heterogeneity (Hédl et al. 2017; Kapfer et al. 2017), which on average increases with area. Thus, we scaled the number of resurvey plots depending on the size of the potential area still covered by grassland (< 10,000 m^2^: 3 plots; 10,000–100,000 m^2^: 4 plots; > 100,000 m^2^: 5 plots). In cases where more than one historical plot shared the same potential area, we sampled at least as many resurvey plots as there were historical plots in the potential area. To avoid autocorrelation, we set a minimum distance between resurvey plots, calculated as the fourth root of the potential area size [m^2^].

#### 2.2.2 Field Sampling

We recorded the resurvey plots in 2021 and 2022 at a similar time of the year as the historical plots to reduce the seasonality bias (Kapfer et al. 2017). Satellite navigation was used to locate each resurvey plot. If the randomly assigned location of the resurvey plot turned out not to be a grassland in the field, or if the total vegetation cover was less than 50%, the plot was relocated according to a predefined protocol. Because it would have been too time‐consuming to sample the plots with the original method (weighted dry biomass for each species), we visually estimated the percent cover of each species using the rooted presence method (Dengler 2008). Due to time constraints, we were not able to resample all historical plots, but we prioritized the sampling to maximize spatial and ecological coverage. In total, we sampled 1107 resurvey plots from 277 potential areas, representing 416 historical plots (Figure 1) over an elevation gradient from 322 to 2497 m. The plant nomenclature of both the historical and the resurvey vegetation records was standardized according to the checklist of the vascular plant flora of Switzerland (Juillerat et al. 2017). Some species that could often not be determined with certainty were grouped into aggregates.

### 2.3 Transformation and Standardisation of Species Abundance

To compare historical and resurvey plots, we transformed the fractional dry biomass into percent cover by applying the allometric regressions that were derived from 40 representative plots sampled with both methods (Riedel et al. 2024) separately for graminoids and forbs. Afterwards, we standardized the estimated cover of the resurvey plots and the transformed biomass weights (~cover) of the historical plots to fractional cover (estimated cover of species/sum of estimated cover × 100).

### 2.4 Diversity Measures

We used species richness as a measure of taxonomic diversity. To compensate for the higher overlooking probability of the resurvey method (on‐site cover estimation in %) compared to the historical method (assessment in the lab, with on average 0.9 more species recorded per plot; Riedel et al. 2024), we added 0.9 to the species richness of each resurvey plot. We used Rao's quadratic entropy index, a generalization of Simpson diversity considering the pairwise differences between species (de Bello et al. 2016), to calculate functional and phylogenetic *α* diversity using the function “melodic” (de Bello et al. 2016). To calculate pairwise additive *β* diversity between pairs of plots, we used the R function “Rao” with Jost correction (de Bello et al. 2010). For the temporal comparison, *β* diversity values for pairs of plots within the same potential area were excluded.

To assess functional diversity, we used the traits “specific leaf area”, “plant height”, and “seed mass”, following the leaf‐height‐seed (LHS) plant strategy scheme (Westoby 1998). Trait data were primarily obtained from the LEDA trait database (Kleyer et al. 2008). Missing values were added from the TRY Global Plant Traits Database (Version 6; Kattge et al. 2020), the Seed Information Database (2023), and Hintze et al. (2013). The values of traits with multiple entries per taxon were averaged. The traits plant height and seed mass were highly skewed and therefore transformed with a logarithm with base 10. Gower distances for species occurring in a plot were then used to calculate Rao's quadratic entropy index.

To compute phylogenetic diversity, we constructed a distance matrix with the phylogenetic distances of all possible species pairs in the species list. For this purpose, we standardized the nomenclature of our species according to the Leipzig catalogue of vascular plants (Freiberg et al. 2020) and then used the function “phylo.maker” of the R package “V.PhyloMaker2” (Jin and Qian 2019) to construct the phylogenetic tree. Using the function “cophenetic” of the package “ape” (Paradis and Schliep 2019), we calculated the phylogenetic distance as the sum of the branch lengths separating two species from each other in the tree. These distances between the species occurring in a plot were then used to calculate Rao's quadratic entropy index.

### 2.5 Ecological Characteristics

We used the ecological indicator values of Landolt et al. (2010) to calculate community‐weighted means for climate (temperature, light) and soil (moisture, reaction, nutrients) variables. In addition, we used the mowing tolerance indicator, which is a proxy for defoliation tolerance, and the indicator for the “influence of man on site condition” (termed hemeroby hereafter). Indicator values for each variable range from 1 (lowest value in Switzerland) to 5 (highest value in Switzerland). We calculated community‐weighted means of the CSR life strategies of plants according to Grime (2001) as provided by Landolt et al. (2010), by decomposing them into the dimensions “competitive ability” (c), “stress tolerance” (s), and “disturbance tolerance” (“ruderal strategist”, r). The values of each dimension were scaled to a range from 0 to 1, with all three combined summing up to 1 for each species (e.g., ssr: *c* = 0, *s* = 2/3, *r* = 1/3). Finally, we also compared the proportions of three important taxonomic groups in grasslands over time: (i) Poaceae, (ii) Cyperaceae and Juncaceae combined, and (iii) forbs (all other families). Cyperaceae and Juncaceae were analyzed separately from Poaceae due to their high abundance in wet grasslands, which have lost most of their former area (Gimmi et al. 2011; Lachat et al. 2010).

### 2.6 Statistical Analyses

We used mixed models with the survey (historical vs. resurvey) as a fixed effect, and the interaction between the potential area and the survey as a random intercept to compare the following response variables between the historical survey and the resurvey: taxonomic, functional, and phylogenetic *α* diversity, mean indicator values (temperature, light, moisture, reaction, nutrients, mowing tolerance, and hemeroby), mean CSR‐values, and proportions of taxonomic groups. For *β* diversity, the potential area pair was used as a random factor. The dependent variables were transformed where necessary to meet model assumptions. The models were fitted using the function “lmer” of the package “lmerTest” (Kuznetsova et al. 2017). All statistical analyses were performed in R version 4.4.1 (R Core Team 2024). To test whether the magnitude of change in an *α* diversity or composition measure varied with elevation, we calculated the potential area mean for the resurvey plots and subtracted the corresponding potential area mean of the historical plots. The resulting difference, e.g., ΔSpecies richness, takes a positive value if the mean species richness was higher in the resurvey than in the historical plots of the potential area and a negative value if the mean species richness of the potential area was higher in the historical than in the resurvey plots. We then used linear models to test whether the difference was related to elevation.

To test the magnitude of *β* diversity change with elevation, we ordered the potential areas by elevation in ascending order and divided them into 11 elevational bands, each containing 25 potential areas except for the first two bands, which contained 26 potential areas to include all 277 potential areas. We then selected all pairwise *β* diversities within an elevational band and used a linear mixed model to compare the two surveys. Finally, we tested whether these differences followed a pattern along the elevational gradient using the median of the elevational band as an independent variable of the linear model. The same approach was used for *γ* diversity. As a proxy for *γ* diversity, we calculated the total species richness of the plots in an elevational band. In order to have the same number of plots in each band for both surveys, we used the function “slice_sample” of the “dplyr” package (Wickham et al. 2023) to randomly select one plot for each potential area and survey. To have an equal number of plots in all elevation bands, we skipped a potential area in the lowest and second lowest bands.

To ascertain that the detected vegetation changes between the two surveys reflect temporal changes and not pseudo‐turnover, we compared the Bray–Curtis dissimilarity among the resurvey plots within a potential area with the average difference between the historical plot(s) and the resurvey plots within that potential area. We calculated Bray–Curtis dissimilarity using the “vegdist” function from the “vegan” package (Oksanen et al. 2024).

## 3 Results

### 3.1 Changes in Diversity

**FIGURE 2:**
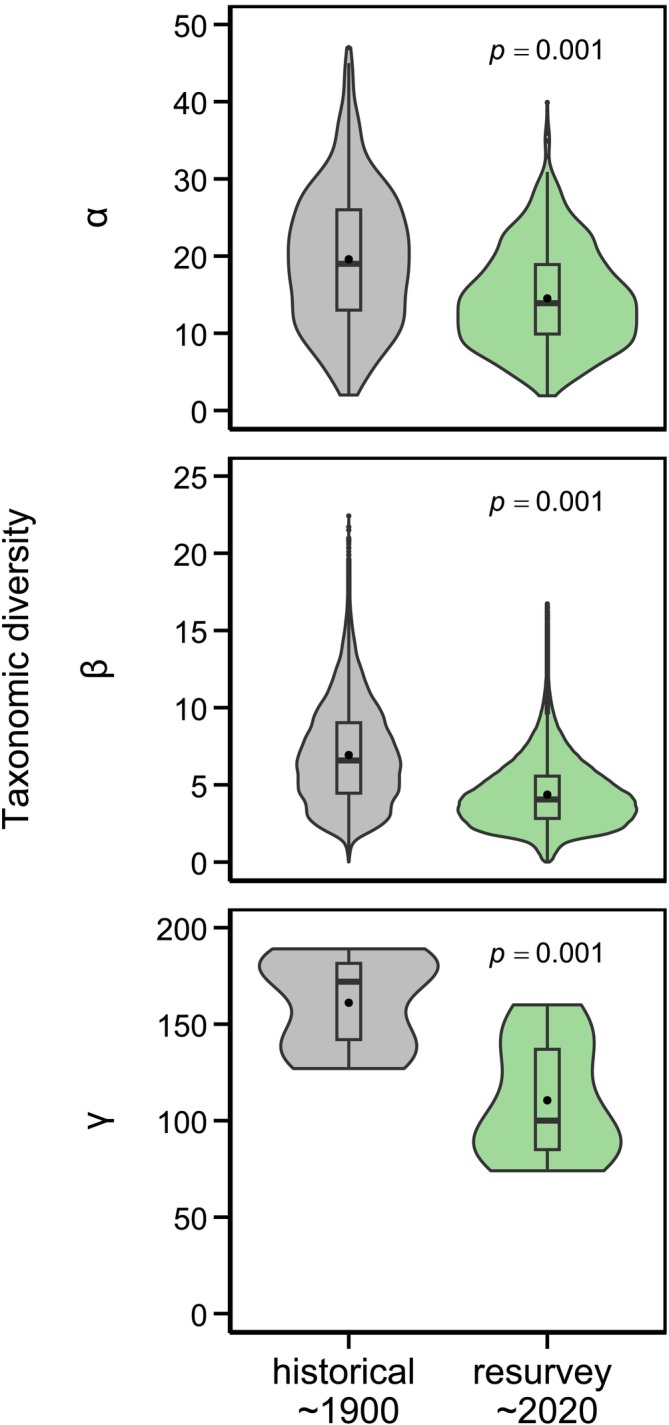
Taxonomic *α* (top), *β* (middle), and *γ* (bottom) diversity were significantly lower in the resurvey plots (2021/2022) than in the historical plots (1884–1931).

Taxonomic diversity decreased at the *α*, *β*, and *γ* levels between the historical survey and the resurvey. Species richness was on average 26% lower in the resurvey plots than in the historical plots (*p* < 0.001). The difference between the resurvey plots and the historical plots (ΔSpecies richness) was largest at low elevations and decreased with increasing elevation (*p* < 0.001). The significant difference in taxonomic *β* (*p* < 0.001) and *γ* (*p* < 0.001) diversity between the historical and the resurvey plots decreased above about 1000 m, reaching a minimum at about 1850 m (Figure 2, Figure S2). Within a given potential area, the mean Bray–Curtis dissimilarity between the historical and the resurvey plots was, on average, 0.13 higher than the dissimilarity between the resurvey plots within the potential area (*p* < 0.001; Figure S1).

**FIGURE 3:**
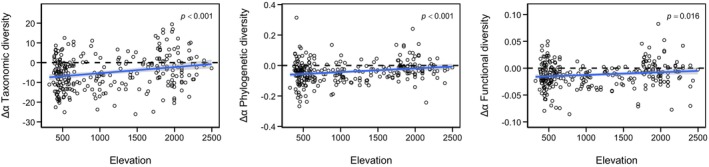
Taxonomic, phylogenetic and functional *α* diversity were significantly lower in the resurvey plots (2021/2022) than in the historical plots (1884–1931). The differences between the two surveys decreased with elevation for taxonomic diversity (Δspecies richness = −8.40 + 0.003 × m, *R* ^2^ adj. = 0.05). Phylogenetic diversity (−0.07 + 2.4e‐05 × m, *R* ^2^ adj. = 0.05) and functional diversity (−0.02 + 5.0e‐06 × m, *R* ^2^ adj. = 0.02).

**FIGURE 4:**
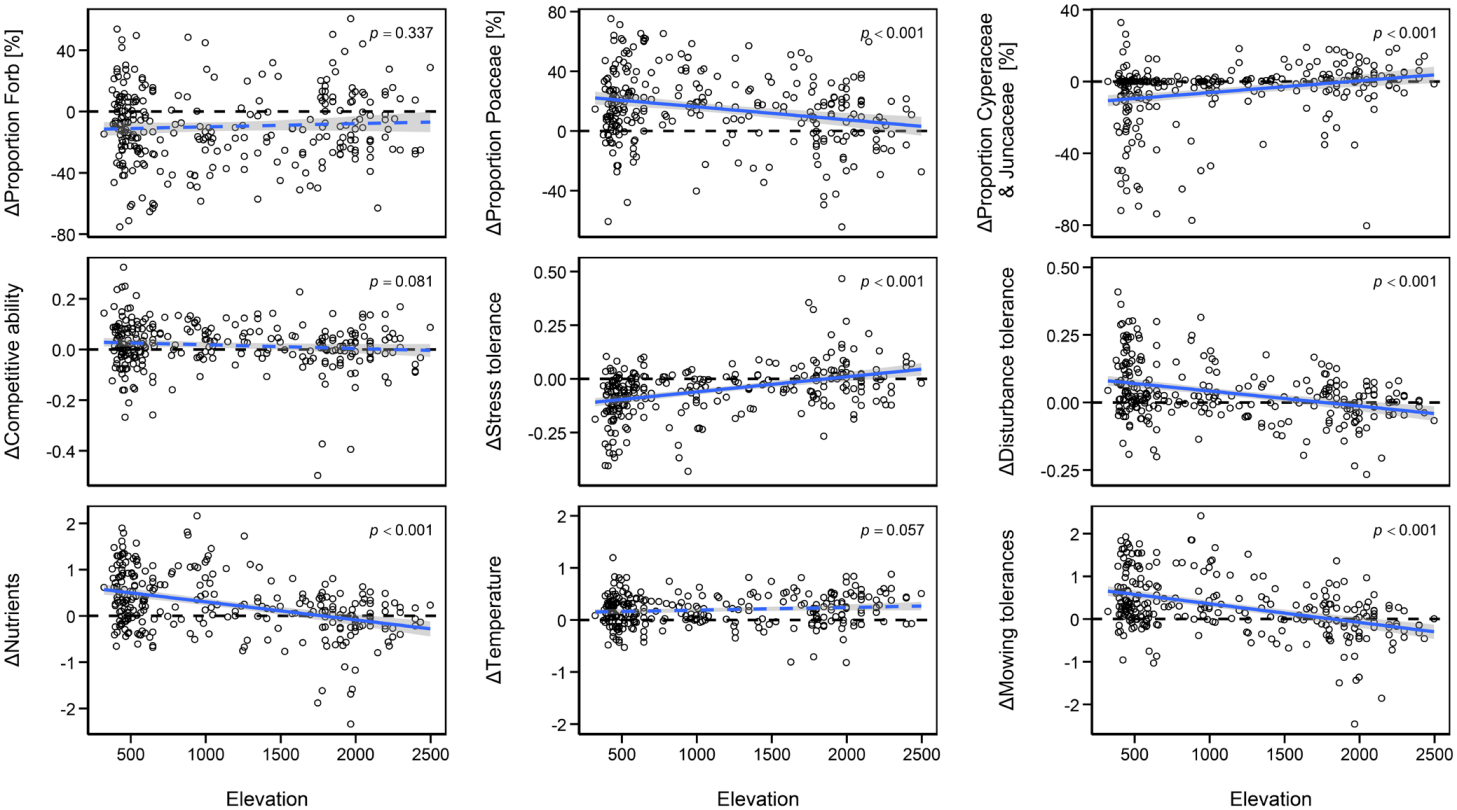
The proportion of Poaceae species was significantly higher in the resurvey plots (2021/2022) than in the historical plots (1884–1931), the differences decreased with increasing elevation (ΔCover Poaceae = 24.9–8.7e‐03 × m, *R* ^2^ adj. = 0.06). In contrast, the proportions of forbs as well as Juncaceae and Cyperaceae were lower in the resurvey plots than in the historical plots. The differences for Cyperaceae and Juncaceae between the two surveys decreased with elevation (−12.78 + 6.6e‐03 × m, *R* ^2^ adj. = 0.06) whereas the magnitude of the change for forbs did not differ with elevation. Stress tolerance was lower in the resurvey plots than in the historical plots up to 1863 m (−0.13 + 7.0e‐05 × m, *R* ^2^ adj. = 0.16). Disturbance tolerance was higher in the resurvey plots up to 1760 m (0.10–5.6e‐05 × m, *R* ^2^ adj. = 0.12). Competitive ability was higher in the resurvey plots than the historical plots; the magnitude of change was not related to elevation. Ecological indicator values for nutrients, mowing tolerance, and temperature were significantly higher in the resurvey plots than in the historical plots. The difference in the indicator value for temperature did not change significantly with elevation. The mean indicator value for nutrients was higher in the resurvey plots than in the historical plots up to 1778 m (0.69–3.9e‐04 × m, *R* ^2^ adj. = 0.15), and the mean indicator value for mowing tolerance up to 1816 m (0.8–4.4e‐04 × m, *R* ^2^ adj. = 0.18).

Phylogenetic *α* diversity (*p* < 0.001) was on average 17% lower in the resurvey plots than in the historical plots, and the difference between the resurvey plots and the historical plots was larger in the lowlands than in the mountains (Figure 3, Figure S3). Phylogenetic *β* diversity (*p* < 0.001) was 23% higher in the resurvey plots than in the historical plots; the difference between the two surveys showed no discernible pattern with elevation (Figure S3). Combined functional *α* diversity (*p* < 0.001; Figure 4, Figure S4) as well as functional *α* diversity for seed mass (*p* < 0.001), plant height (*p* < 0.001), and specific leaf area (*p* < 0.001) separately, was significantly lower in the resurvey than in the historical survey (Figure S5). Functional *β* diversity was 4% higher (*p* < 0.001) in the resurvey plots than in the historical plots; the difference between the two surveys was not related to elevation (Figure S4).

### 3.2 Changes in Community Characteristics

The resurvey plots had on average 47% more Poaceae species cover than the historical plots (*p* < 0.001), but 17% less forbs cover (*p* < 0.001) and 42% less Cyperaceae and Juncaceae cover (*p* < 0.001). The proportion of Poaceae increased more strongly in the lowlands than at high elevations (*p* < 0.001). Conversely, Cyperaceae and Juncaceae decreased more strongly in the lowlands than in the mountains (*p* < 0.001). Forbs declined at a similar rate along the entire elevational gradient (*p* = 0.337; Figure 4, Figure S6).

Disturbance tolerance (*p* < 0.001) and competitive ability (*p* = 0.002) were higher in the resurvey plots than in the historical plots, whereas stress tolerance was lower in the resurvey plots than in the historical plots (*p* < 0.001). The difference in stress tolerance decreased with elevation, and stress tolerance was similar for both surveys at about 1850 m (*p* < 0.001). Disturbance tolerance was higher in the resurvey plots up to about 1750 m (*p* < 0.001). The magnitude of change in competitive ability was not related to elevation (*p* = 0.081; Figure 4, Figure S7).

The community‐weighted means of the ecological indicator values for nutrients (*p* < 0.001), temperature (*p* < 0.001), mowing tolerance (*p* < 0.001), and hemeroby (*p* < 0.001) were significantly higher in the resurvey plots than in the historical plots. The mean indicator value for hemeroby was higher in the resurvey plots along the whole elevational gradient, whereas the mean indicator values for nutrients and mowing tolerance were higher in the resurvey plots than in the historical plots up to about 1800 m, above which they were lower in the resurvey plots than in the historical plots. The mean temperature indicator values increased at a similar rate along the elevational gradient. The mean indicator values for reaction were significantly lower in the resurvey plots (*p* = 0.010), whereas no significant difference was found between the historical and the resurvey plots for light (*p* = 0.991) and moisture (*p* = 0.092; Figure 4, Figure S8).

## 4 Discussion

Our study provides the first nationwide, century‐scale resurvey of semi‐natural grasslands using species‐level community data. We document a consistent decline in *α*, *β*, and *γ* species diversity in Switzerland, reflecting widespread ecological shifts linked to long‐term land‐use intensification and environmental change.

### 4.1 Taxonomic, Functional, and Phylogenetic Diversity Loss

Average local species richness (*α* diversity) declined by over a quarter. A decrease in species richness in semi‐natural grassland has also been found in other resurvey studies, particularly those in which the historical survey had been conducted in the 1960s or earlier (Bennie et al. 2006; Homburger and Hofer 2012; McGovern et al. 2011; Ross et al. 2012; Wesche et al. 2012). In contrast, resurvey studies using historical data sampled after the 1960s have often found no significant change (Koch et al. 2017) or even an increase in species richness (Britton et al. 2005; Gillet et al. 2016; Peter et al. 2008, 2009; Schwaiger et al. 2022). These contrasting findings are probably because the most drastic land use intensification in Central Europe took place in the 1950s and 1960s, even though grassland diversity has a relatively long lag time after management change (Helm et al. 2005). Accordingly, studies in Germany have found that the strongest decline in native plant species richness occurred in 1960–1980 (Eichenberg et al. 2021; Jandt, Bruelheide, Jansen, et al. 2022).

Apart from the loss of *α* diversity (species richness), we also demonstrated that Swiss grasslands experienced a loss in *β* diversity, leading to taxonomic homogenization. Taxonomic homogenization, though frequently concurrent with biodiversity loss, is not necessarily contingent upon declines in *α* diversity (Olden 2006; Olden and Rooney 2006). It may occur under scenarios of reduced, stable, or even increasing local species richness (Britton et al. 2009; Liberati et al. 2019). For instance, an earlier resurvey of Swiss grasslands between 2001–2004 and 2006–2009 documented rising species richness alongside declining *β* diversity, attributable to the increasing dominance of widespread generalist species (Bühler and Roth 2011). These divergent outcomes likely reflect the temporal scale of analysis: short‐term resurveys often capture transient dynamics or early successional changes, whereas our century‐scale perspective reveals long‐term trends driven by cumulative anthropogenic pressures. The pronounced decline in *β* diversity observed in our study is best understood in the context of progressive, large‐scale land‐use intensification since the early 20th century. While moderate increases in land‐use intensity have been shown to drive homogenization across taxa and ecosystems (Gossner et al. 2016), the rapid and widespread transformation of European lowland agriculture—peaking in the mid‐20th century—likely accelerated this process in Swiss grasslands. As described for *α* diversity, the most intense shifts probably occurred during and shortly after this period of peak intensification.

In addition to *α* and *β* diversity losses, we observed a notable decline in *γ* diversity, quantified as total species richness across elevational bands. This reduction in regional‐scale diversity aligns with findings from historical grassland resurveys in Germany, which similarly reported substantial *γ* diversity loss over multi‐decadal timescales (Wesche et al. 2012). Supporting evidence at the national scale indicates declining abundances of specialist species associated with nutrient‐poor grasslands across Switzerland during the 20th century (Bosshard 2015; Scherrer et al. 2022). Together, these patterns underscore a pervasive erosion of plant diversity at all spatial scales—reflecting a broad‐scale restructuring of grassland floras driven primarily by land‐use intensification, with implications for regional ecosystem stability and biotic distinctiveness under ongoing global change.

In agreement with our hypothesis, we found that both functional and phylogenetic *α* diversity declined in contemporary plots compared to historical baselines. Although these declines were less pronounced than those in taxonomic *α* diversity, they likely reflect parallel processes: reduced species richness constrains the diversity of both traits and evolutionary lineages (Abrahamczyk et al. 2020; de Bello et al. 2016). However, in contrast to taxonomic *β* diversity, which declined over time, functional and phylogenetic *β* diversity increased. This may reflect historically higher functional redundancy, with more species occupying similar niches (Mayfield et al. 2010). For instance, moisture‐tolerant Juncaceae species were more widespread a century ago, likely due to lower drainage intensity at lower elevations (Gimmi et al. 2011; Lachat et al. 2010). The loss of such functionally similar species can elevate functional *β* diversity, as these indices are sensitive to trait differentiation among remaining species (Olden 2006). The less pronounced decline in *α* functional diversity than in species richness suggests greater historical redundancy at broader spatial scales. Given our small plot sizes, much of the diversity likely existed among rather than within plots, underscoring the scale dependence of diversity patterns (Chase et al. 2019). Functional redundancy is vital for ecosystem resilience, enabling compensation when species are lost (McCann 2000). Its decline in Swiss grasslands implies increasing vulnerability to environmental change. Similar trends in German grasslands (Wesche et al. 2012) point to widespread impacts of long‐term anthropogenic pressures.

The observed decline in phylogenetic diversity suggests a loss of evolutionary distinctiveness. Since functional traits often exhibit phylogenetic conservatism (de Bello et al. 2017; Kraft et al. 2007), similar trends are expected (but see Večeřa et al. 2023). Phylogenetic diversity also captures unmeasured functional variation (de Bello et al. 2015, 2017), such as chemical or root traits, providing important complementary information. Comparable declines in functional and phylogenetic *α* diversity were noted in grasslands of the French Jura Mountains over two decades (Gillet et al. 2016), though species richness slightly increased. Such discrepancies highlight the context‐dependence of diversity change. However, few long‐term resurvey studies incorporated functional or phylogenetic metrics to date, limiting general conclusions. Expanding such studies remains essential to understand and manage biodiversity under global change.

### 4.2 Ecological Filtering and Community Shifts

Our results show a marked increase in the proportion of Poaceae species in the resurvey plots, parallel to a decrease in the proportion of all other plant groups—a pattern consistent with numerous grassland resurvey studies at plot (Bauer and Albrecht 2020; Berlin et al. 2000; Gillet et al. 2016; McGovern et al. 2011) and regional scale (Abrahamczyk et al. 2022; Wesche et al. 2012). As Poaceae are wind‐pollinated, this shift increased the prevalence of wind‐pollinated species and may thus reduce habitat quality, particularly for specialist pollinators (Biesmeijer et al. 2006). The increased dominance of Poaceae is likely linked to increased mowing and grazing intensity, as many species in this family are specifically adapted to such repeated biomass removal via traits such as basal meristems or clonal growth (Díaz et al. 2007; Hawkes and Sullivan 2001). Moreover, Poaceae are known to react positively to increased nitrogen availability (Duprè et al. 2010).

Life strategy analyses further revealed a shift toward species with greater competitive ability and disturbance tolerance, but lower stress tolerance in resurvey plots. This pattern suggests a directional shift along Grime's CSR axis, likely driven by nutrient enrichment and warming temperatures (Isotta et al. 2019), Higher mowing and grazing frequency likely promoted disturbance‐tolerant species, although closed swards in intensively managed grasslands could limit opportunities for ruderal establishment (Wesche et al. 2012). Nevertheless, mechanical practices like slurry application and higher stocking rates may have offset this by maintaining open microsites (Rook et al. 2004).

Ecological indicator values support these findings. Increases in mean indicator values for nutrients, mowing tolerance, and hemeroby reflect intensified land use and elevated nitrogen deposition—now roughly double the estimated 1880 levels (Rihm 2023; Roth et al. 2015). This trend mirrors findings from numerous European resurvey studies (Bauer and Albrecht 2020; Bennie et al. 2006; Diekmann et al. 2019; Gillet et al. 2016; Klinkovská et al. 2024; Peter et al. 2008; Stevens et al. 2016; Wesche et al. 2012), Switzerland's agricultural nitrogen and phosphorus surpluses further corroborate this shift (FSO 2024a, 2024b). Consistent with previous studies, higher nitrogen availability is associated with declining species richness (Billeter et al. 2008; De Schrijver et al. 2011; Kleijn et al. 2009; Roth et al. 2013). We found no significant change in the mean indicator value for light, possibly due to frequent biomass removal through mowing and grazing, which may maintain light availability for subordinate species (Borer et al. 2014). The observed increase in temperature indicator values was weaker than expected considering the average temperature increase in Switzerland of around 2°C since 1864 (CH2018. 2018; Isotta et al. 2019). Based on the observed shift in mean temperature indicator values along the elevation gradient in Switzerland (−0.098 per 100 m; Kiebacher et al. 2023) and the lapse rate of −0.65°C per 100 m (Meteo Swiss 2025), a temperature increase of 2°C should lead to an increase of around 0.3 in the mean ecological indicator value for temperature. This is about 50% higher than what we found (0.19). This is possibly due to species turnover lag or stable cold‐adapted populations (BAFU 2017). The significant but only slight decrease in reaction indicator values could be related to a partial recovery from the effect of acid rain after a reduction of acid deposition in the last decades and liming practices (Flisch et al. 2017; Rose et al. 2016). Finally, moisture indicator values remained unchanged, despite known wetland drainage (Gimmi et al. 2011). This may be due to offsetting factors, such as irrigation or denser swards that increase microclimatic humidity (Charmillot et al. 2021), or because wet grasslands were underrepresented in the historical sample (Riedel et al. 2023).

### 4.3 Elevation‐Dependent Patterns of Change

Differences in diversity and ecological characteristics between historical and resurvey plots were linearly related to elevation (Table S1), with stronger biodiversity loss and compositional shifts at lower elevations (colline to montane belts) than at higher ones (subalpine to alpine belts). This pattern likely reflects stronger land‐use intensification in accessible lowland sites, where mechanization, fertilization, drainage, and re‐sowing have enabled more frequent mowing and higher livestock densities (Ceulemans et al. 2013, 2014; Dengler et al. 2020). In contrast, grasslands at higher elevations—typically steeper and less accessible—have been less intensively managed due to topographic and climatic constraints (Landoldt and Urbanska 2003).

In addition to direct land use, indirect drivers such as nitrogen deposition and climate change have altered abiotic conditions. Atmospheric nitrogen deposition in Switzerland ranges from 2 to 43 kg N ha^−1^ year^−1^, far above natural levels (~1 kg N ha^−1^ year^−1^; Butterbach‐Bahl et al. 2011), with highest values in lowland valleys (Rihm 2023). This explains why the mean indicator value for nutrients increased more below ~1800 m, reflecting both fertilization and atmospheric deposition. The diversity impact of nitrogen depends on climate: its effect is weaker in mountain areas with cool summers than in warmer ones (Humbert et al. 2016), suggesting increasing vulnerability of subalpine grasslands with climate warming.

Temperatures have risen at all elevations over the last 150 years, with more pronounced summer warming in the Alps (Isotta et al. 2019). Precipitation trends are less clear, though extremes are increasing (Scherrer et al. 2016). While warming can drive upward shifts in plant optima (Lenoir et al. 2008; Roth et al. 2014; Vitasse et al. 2021), we found no elevation‐dependent change in the temperature indicator value, suggesting that climate change has so far had less impact on grassland diversity than land use and eutrophication.

The environmental changes described here—particularly the stronger intensification at lower elevations—have altered environmental filtering processes (Mayfield et al. 2010) and the regional species pool (Olde Venterink 2011), favoring competitive, nutrient‐demanding species like Poaceae. This has reshaped the elevation–diversity gradient, such that today's patterns no longer reflect historical baselines (Nogués‐Bravo et al. 2008). The elevation dependence of biodiversity loss underscores the need for elevation‐sensitive conservation. For instance, protected areas have helped to maintain specialist species below 1000 m, where the protected areas are often islands surrounded by intensively managed land, but not above where land use is less intense (Dähler et al. 2019).

## 5 Conclusions

We demonstrate a systematic decline in *α*, *β*, and *γ* taxonomic diversity across Swiss grasslands over the past century. On average, *α* diversity decreased by 26% and *γ* diversity by 31% compared to the historical baseline, indicating substantial biodiversity loss. In contrast, recent data from the Swiss national biodiversity monitoring program show that grassland *α* diversity has increased over the past two decades, while *β* and *γ* diversity remained stable (Häberlin and Dengler 2025). This contrast underscores the limitations of short‐term resurvey studies and highlights the critical value of long‐term baselines in understanding biodiversity trends. The much greater decline in diversity and ecological indicators at lower elevations compared to higher ones—for example, a 38% species loss at 500 m vs. 11% at 2000 m—points to the major role of land‐use intensification and nitrogen input and deposition. This suggests that the current diversity–elevation gradient is heavily shaped by anthropogenic influence. Conservation efforts at lower elevations can still be impactful, especially in preserving specialised species (Dähler et al. 2019), while the relatively smaller losses in higher‐elevation grasslands offer a valuable opportunity to conserve much of the historical plant diversity (Kampmann et al. 2012). However, these mountain refuges face increasing threats from climate change, which may facilitate an upward shift of land‐use intensification. This could accelerate the trend of intensifying more accessible, productive alpine grasslands while more remote areas are abandoned (Herzog and Seidl 2018), putting remaining high‐elevation biodiversity at risk.

## Author Contributions

**Stefan Widmer:** data curation, formal analysis, investigation, methodology, visualization, writing – original draft, writing – review and editing. **Susanne Riedel:** data curation, investigation, writing – review and editing. **Manuel Babbi:** data curation, investigation, writing – review and editing. **Felix Herzog:** funding acquisition, writing – review and editing. **Thomas Wohlgemuth:** writing – review and editing. **Michael Kessler:** conceptualization, methodology, writing – review and editing. **Jürgen Dengler:** conceptualization, funding acquisition, methodology, writing – review and editing.

## Conflicts of Interest

The authors declare no conflicts of interest.

## Supporting information


**Data S1:** gcb70529‐sup‐0001‐Supinfo.pdf.

## Data Availability

The data that support the findings of this study are openly available in figshare at http://doi.org/10.6084/m9.figshare.27170790.
