# Supplementary material for: One Century of Change: Stronger Diversity Decline in Lowland Than in Mountain Grasslands in Central Europe
Source: Glob Chang Biol. 2025 Oct 3;31(10):e70529. doi: 10.1111/gcb.70529 (PMC12492779; doi:10.1111/gcb.70529)
Supplement: Supplementary file 1 — Data S1: gcb70529‐sup‐0001‐Supinfo.pdf. [file GCB-31-e70529-s001.pdf]

Global Change Biology, 2025

One century of change: stronger diversity decline in lowland than in mountain grasslands in Central Europe

Stefan Widmer[1,2], Susanne Riedel[2,3], Manuel Babbi[1], Felix Herzog[3], Thomas Wohlgemuth[4], Michael Kessler[2,*], Jürgen Dengler[1,*]

[1] Vegetation Ecology Research Group, Institute of Natural Resource Sciences, ZHAW Zurich University of Applied Sciences, Wädenswil, Switzerland

[2] Department of Systematic and Evolutionary Botany, University of Zurich, Zurich, Switzerland

[3] Agricultural Landscapes and Biodiversity, Agroscope, Zurich, Switzerland

[4] Swiss Federal Institute for Forest, Snow and Landscape Research WSL, Birmensdorf, Switzerland

* Jürgen Dengler and Michael Kessler shall be considered joint senior authors.

Correspondence

Stefan Widmer, Vegetation Ecology Research Group, Institute of Natural Resource Sciences, ZHAW Zurich University of Applied Sciences, Grüentalstr. 14, 8820 Wädenswil, Switzerland, Email: stefan.widmer@zhaw.ch

Author ORCIDs

Stefan Widmer: https://orcid.org/0000-0002-4920-5205
Susanne Riedel: https://orcid.org/0000-0003-0471-2307
Felix Herzog: https://orcid.org/0000-0001-9472-4891
Thomas Wohlgemuth: https://orcid.org/0000-0002-4623-0894
Michael Kessler: https://orcid.org/0000-0003-4612-9937
Jürgen Dengler: https://orcid.org/0000-0003-3221-660X

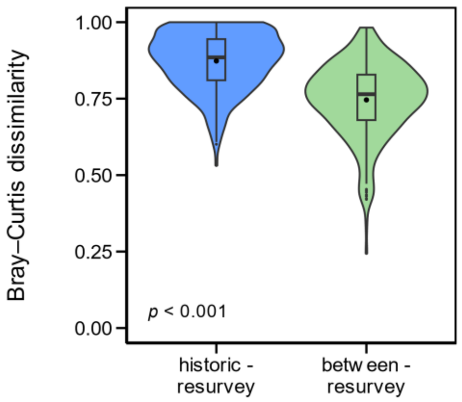

Fig. S1: Comparison of the mean Bray-Curtis dissimilarity between the historical and the corresponding resurvey plots within one potential area and the mean dissimilarity between the resurvey plots within one potential area. The mean Bray-Curtis dissimilarity between the historical and the resurvey plots was 0.13 higher than the mean dissimilarity between the resurvey plots in one potential area (p < 0.001).

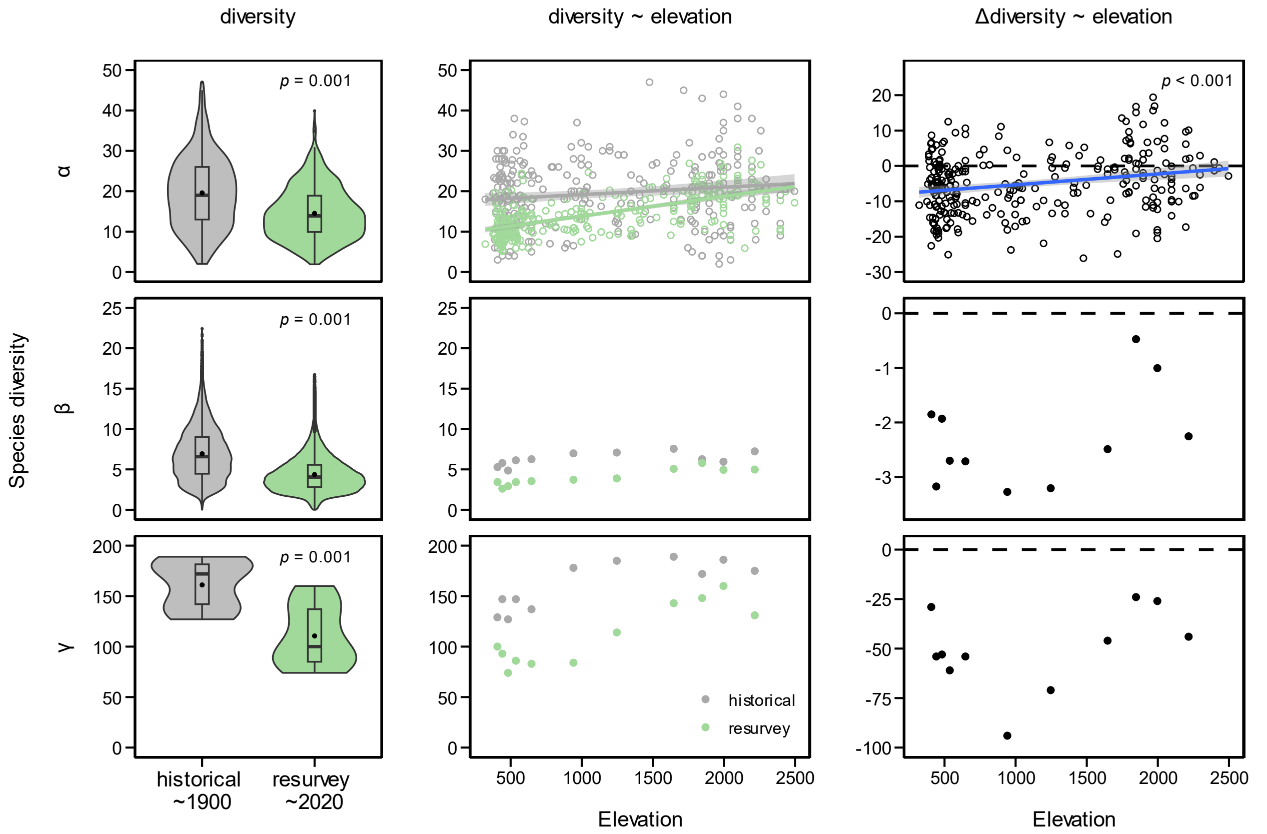

Fig. S2 Taxonomic α (top), β (middle), and γ (bottom) diversity were significantly lower in the resurvey plots (2021/2022) than in the historical plots (1884–1931). The difference in species richness (species richness resurvey plot – species richness historical plot) between the historical survey and the resurvey decreased with elevation (Δspecies richness = -8.40 + 0.003 × m, R^2 adj. = 0.05). β und γ taxonomic diversity showed the greatest difference between historical and resurvey plots around 1000 m. The difference in β diversity was significant (p <0.001) for all elevational bands.

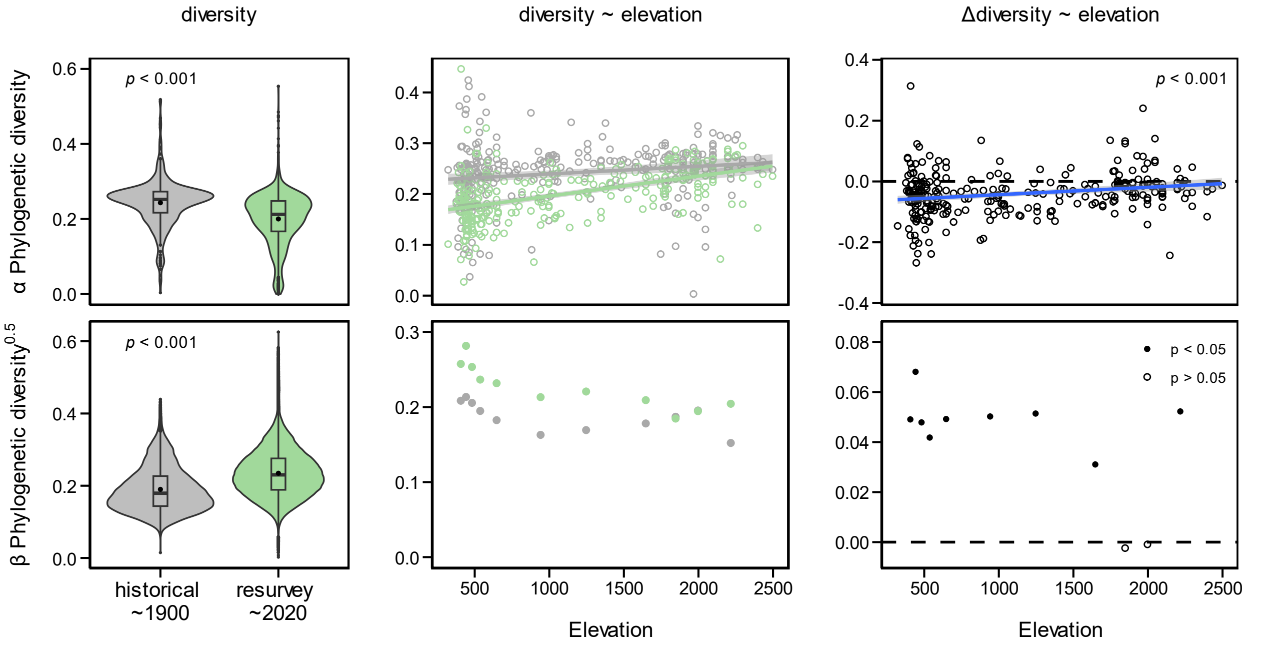

Fig. S3: Phylogenetic α diversity (top) was significantly lower in the resurvey plots (2021/2022) than in the historical plots (1884–1931). The differences between the two surveys decreased with elevation (-0.07 + 2.4e-05 × m, R^2 adj. = 0.05). Phylogenetic β diversity (bottom) was higher in the resurvey than in the historical plots. Differences in β diversity showed no discernible relationship with elevation.

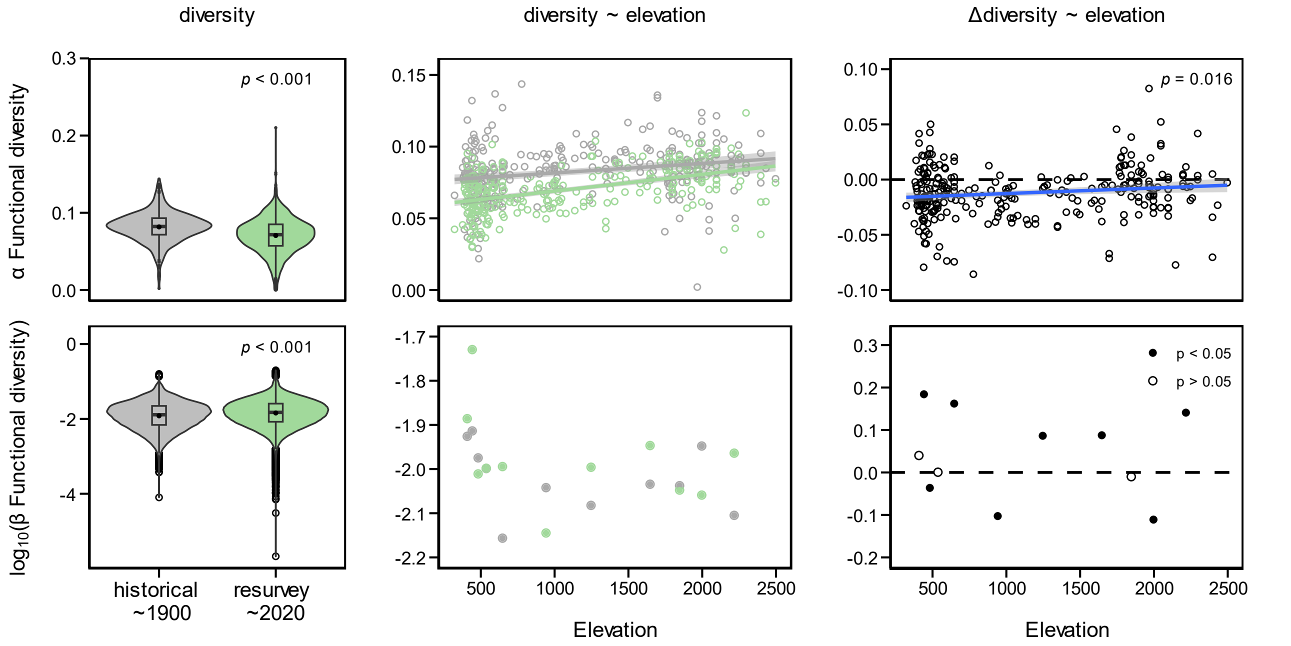

Fig. S4: Functional α diversity (top) was significantly lower in the resurvey plots (2021/2022) than in the historical plots (1884–1931). The differences between the two surveys decreased with elevation (-0.02 + 5.0e-06 × m, R^2 adj. = 0.02). Functional β diversity (bottom) was higher in the resurvey than in the historical plots. Differences in β diversity showed no discernible relationship with elevation.

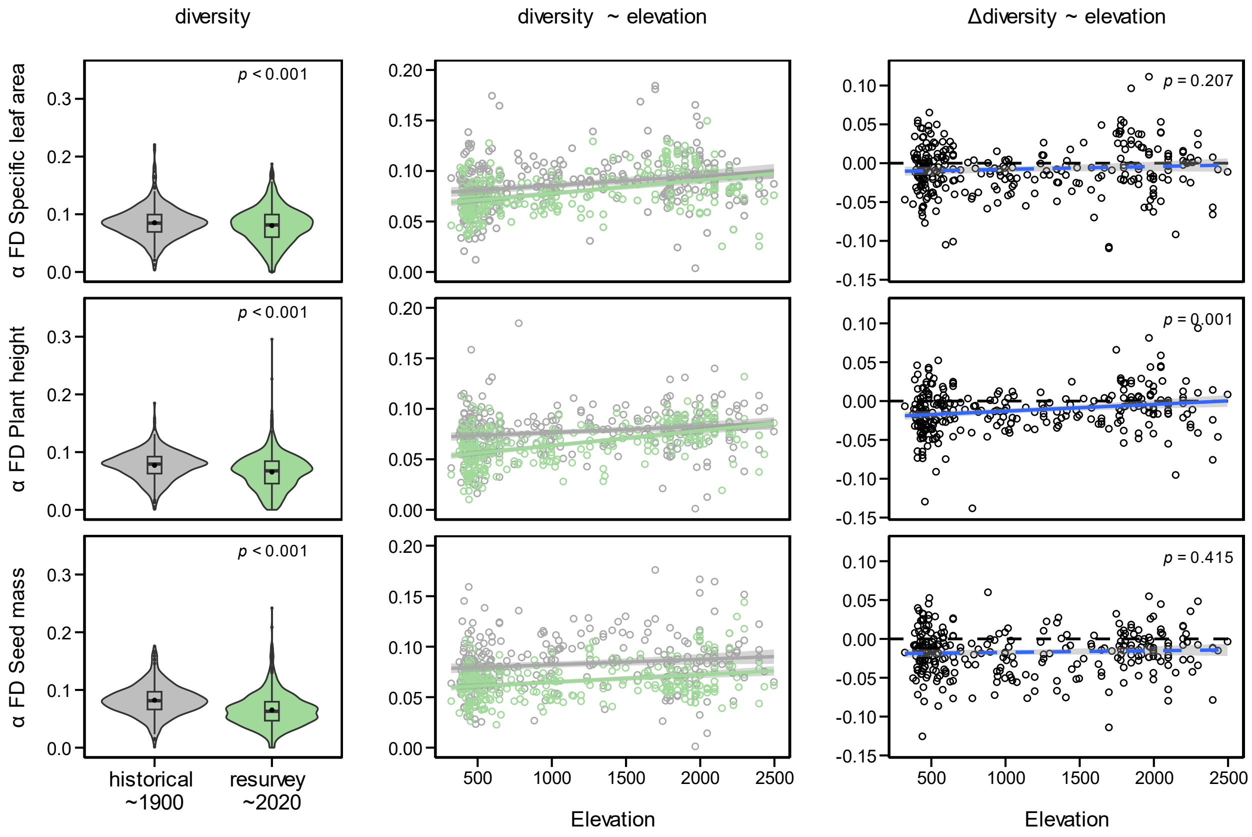

Fig. S5: Functional diversity (FD) for specific leaf area, plant height and seed mass were significantly lower in the resurvey (2021/2022) than in the historical survey (1884–1931). The difference for functional diversity for plant height decreased with increasing elevation (-0.02 + 8.7e-06 × m, R^2 adj. = 0.04), whereas the magnitude of change of the functional diversity for specific leaf area and seed mass did not significantly change with elevation.

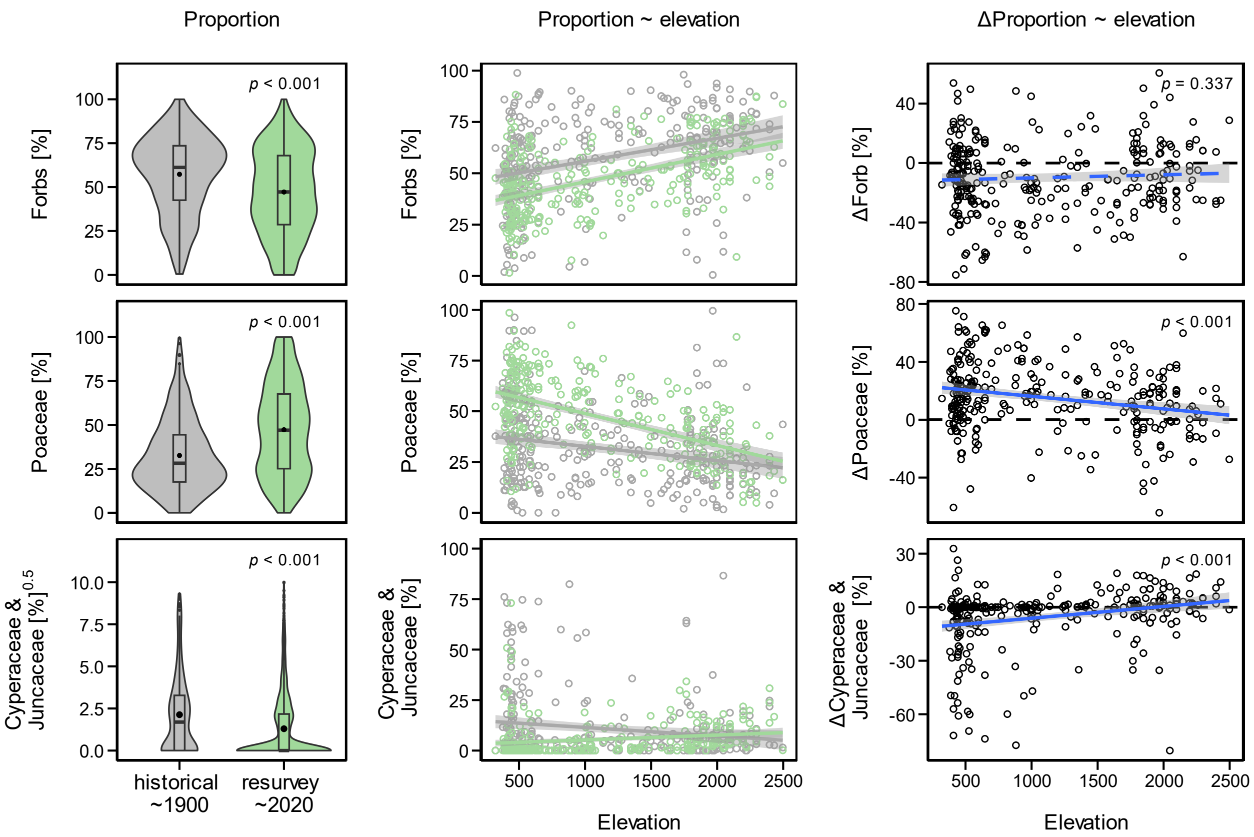

Fig. S6: The proportion of Poaceae species was significantly higher in the resurvey plots (2021/2022) than in the historical plots (1884–1931), and the differences decreased with increasing elevation (24.9 - 8.7e-03 × m, R^2 adj. = 0.06). In contrast, the proportions of forbs as well as Juncaceae and Cyperaceae were lower in the resurvey plots than in the historical plots. The differences for Cyperaceae and Juncaceae between the two surveys decreased with elevation (-12.78 + 6.6e-03 × m, R^2 adj. = 0.06) whereas the magnitude of the change for forbs did not differ with elevation.

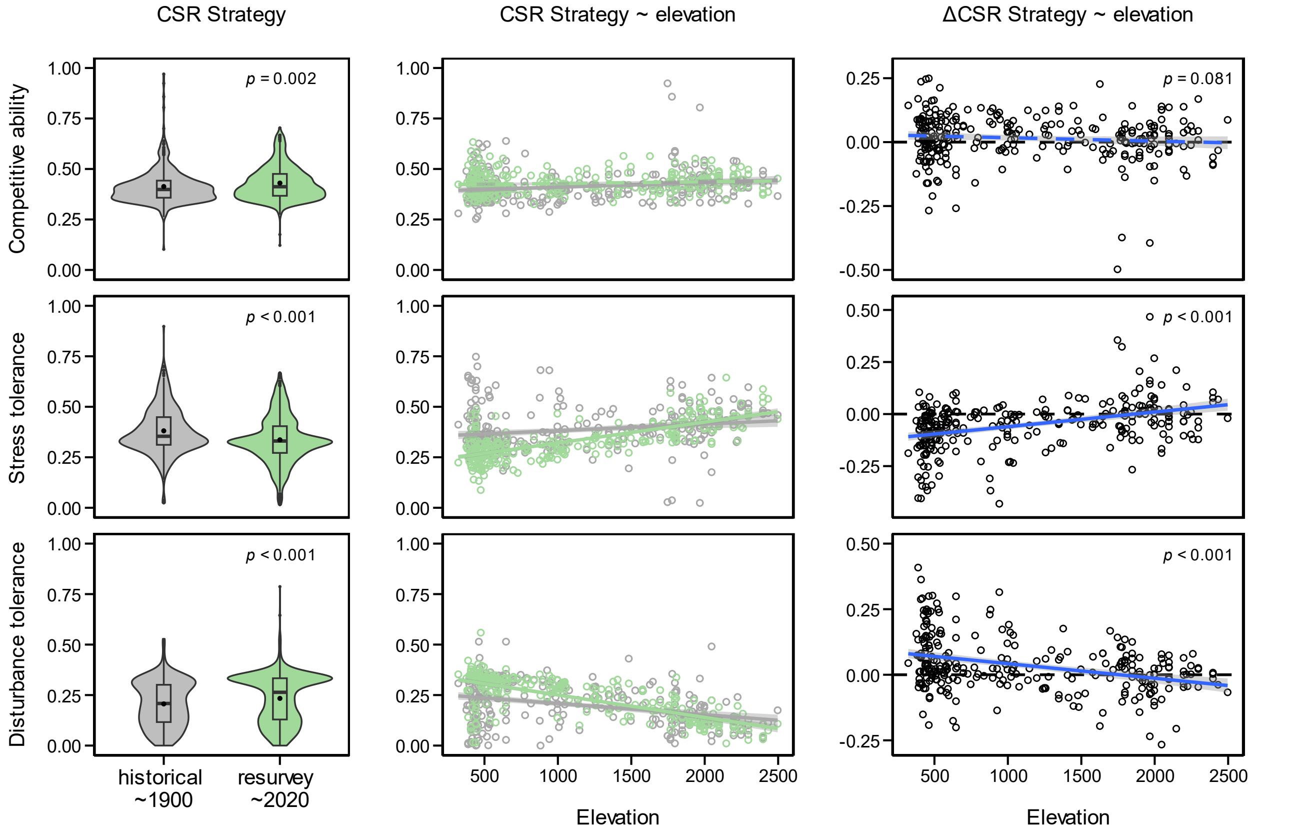

Fig. S7: Stress tolerance was lower in the resurvey plots (2021/2022), than in the historical plots (1884–1931), up to 1861 m (-0.13 + 7.0e-05 × m, R^2 adj. = 0.16). Disturbance tolerance was higher in the resurvey plots up to 1760 m (0.10 + 5.6e-05 × m, R^2 adj. = 0.12). Competitive ability was higher in the resurvey plots than the historical plots the magnitude of change was not related to elevation.

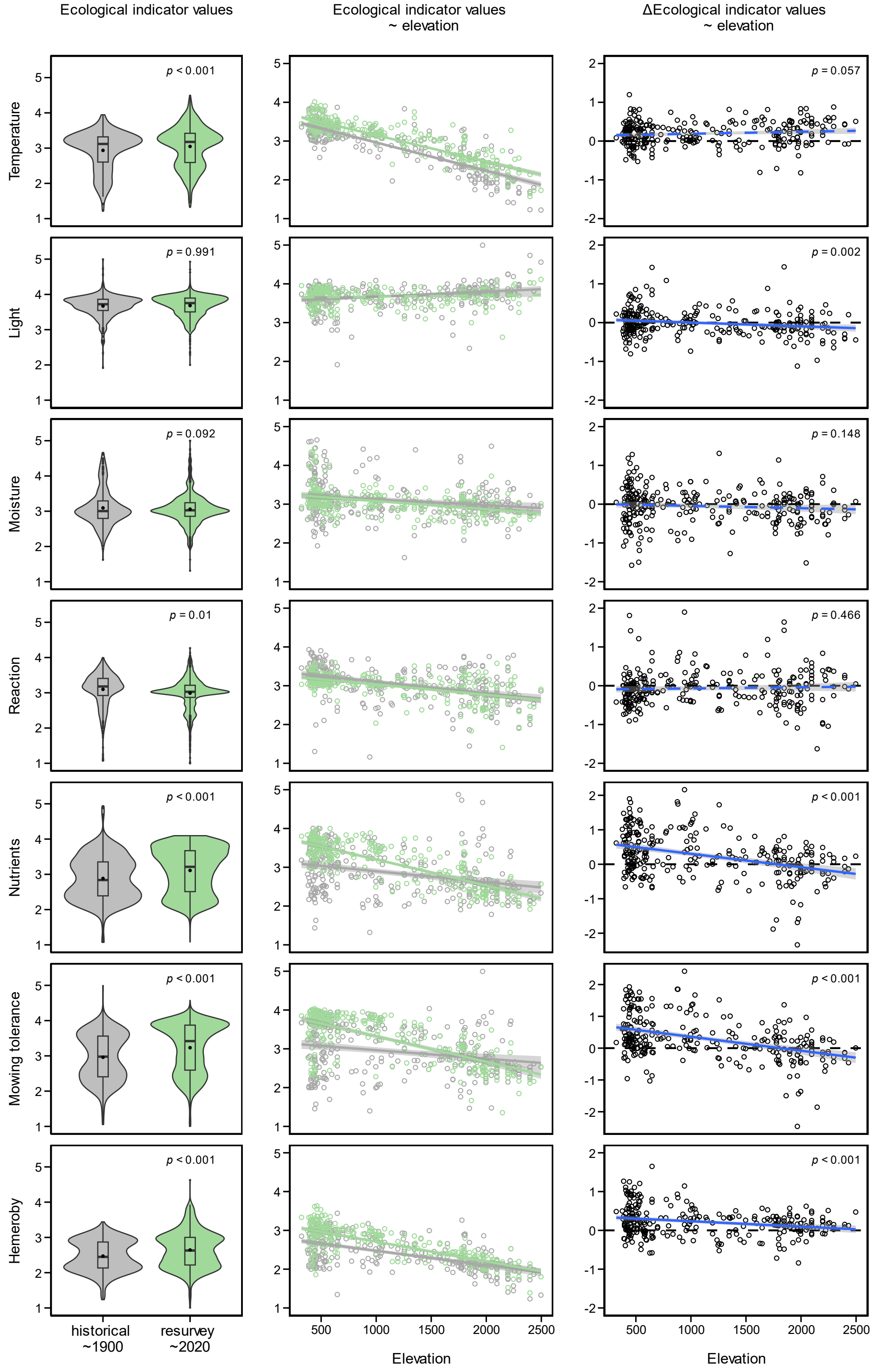

Fig. S8: The community-weighted mean ecological indicator values for temperature, nutrients, mowing tolerance and hemeroby were significantly higher in the resurvey plots (2021/2022) than in the historical plots (1884–1931). The difference in the mean indicator value for temperature did not change significantly with elevation. The mean indicator value for nutrients was higher in the resurvey plots than in the historical plot up to 1778 m (0.69 -3.9e-04 × m, R^2 adj. = 0.15), the indicator value for mowing tolerance up to 1816 m (0.8 -4.4e-04 × m, R^2 adj. = 0.18). For hemeroby, the difference between the two surveys decreased with increasing elevation (0.4 -1.4e-04 × m, R^2 adj. = 0.06). The mean indicator value for reaction was significantly lower in resurvey plots than on the historical plots, but the difference was not linearly related to elevation. The mean indicator value for light overall was not significantly different between the surveys, below 1017 m the value was higher in the resurvey plot above 1017 m the value was higher for the historical plots (0.1 - 9.7e-05 × m, R^2 adj. = 0.03). The difference in moisture was overall not significant between the two surveys and was not significantly related to elevation.

Table S1: Linear models with the species diversity and composition delta measures (mean resurvey plots – mean historical plots) as dependent variable and the elevation as independent variable. In case of a significant elevation-dependence, the predicted elevation of zero change is given. FD = Functional diversity; EIV = ecological indicator value.

	**Intercept**	**Elevation**	**Adj. R^2**	***p***	**Δ = 0 [m]**
ΔSpecies richness	-8.40	0.003061	0.05	<0.001	2745
ΔPhylogenetic diversity	-0.07	0.000024	0.05	<0.001	2797
ΔFunctional diversity	-0.02	0.000005	0.02	0.016	3516
ΔFD Seed mass	-0.02	0.000002	0.00	0.415	n.s.
ΔFD Plant height	-0.02	0.000009	0.04	0.001	2483
ΔFD Specific leaf area	-0.01	0.000004	0.00	0.207	n.s.
ΔCover Forb	-12.13	0.002112	0.00	0.337	n.s.
ΔCover Poaceae	24.91	-0.008713	0.06	<0.001	2859
ΔCover Cyperaceae & Junceae	-12.78	0.006601	0.06	<0.001	1936
ΔCSR Competitive ability	0.03	-0.000015	0.01	0.081	n.s.
ΔCSR Stress tolerance	-0.13	0.000071	0.16	<0.001	1861
ΔCSR Disturbance tolerance	0.10	-0.000056	0.12	<0.001	1760
ΔEIV Temperature	0.14	0.000051	0.01	0.057	2663
ΔEIV Light	0.10	-0.000097	0.03	0.002	1017
ΔEIV Moisture	0.02	-0.000059	0.00	0.148	n.s.
ΔEIV Reaction	-0.10	0.000030	0.00	0.466	n.s.
ΔEIV Nutrient	0.69	-0.000389	0.15	<0.001	1778
ΔEIV Hemeroby	0.37	-0.000137	0.06	<0.001	2712
ΔEIV Mowing tolerance	0.80	-0.000438	0.18	<0.001	1816
